# Threshold protective levels of serum IgG to *Shigella* lipopolysaccharide: re-analysis of *Shigella* vaccine trials data

**DOI:** 10.1016/j.cmi.2022.10.011

**Published:** 2023-03

**Authors:** Dani Cohen, Shai Ashkenazi, Rachel Schneerson, Nahid Farzam, Anya Bialik, Shiri Meron-Sudai, Valeria Asato, Sophy Goren, Tomer Ziv Baran, Khitam Muhsen, Peter B. Gilbert, Calman A. MacLennan

**Affiliations:** 1) Department of Epidemiology and Preventive Medicine, School of Public Health, Sackler Faculty of Medicine, Tel Aviv University, Tel Aviv, Israel; 2) Adelson School of Medicine, Ariel University, Ariel, Israel; 3) Schneider Children's Medical Center, Petach Tikva, Israel; 4) National Institute of Child Health and Human Development, National Institutes of Health, Bethesda, MD, USA; 5) Division of Endocrinology, Diabetes and Metabolism, Sheba Medical Center, Tel Hashomer, Ramat Gan, Tel Aviv, Israel; 6) Vaccine and Infectious Disease Division, Fred Hutchinson Cancer Research Center, Seattle, WA, USA; 7) Department of Biostatistics, University of Washington, Seattle, WA, USA; 8) Bill and Melinda Gates Foundation, London, United Kingdom; 9) Nuffield Department of Medicine, Jenner Institute, University of Oxford, Oxford, United Kingdom

**Keywords:** Conjugate vaccines, Correlate of protection, Immunological substitute, Serum IgG, Shigella, Vaccine efficacy

## Abstract

**Objectives:**

Establishing a correlate of protection is essential for the development and licensure of *Shigella* vaccines. We examined potential threshold levels of serum IgG to *Shigella* lipopolysaccharide (LPS) that could predict protection against shigellosis.

**Methods:**

We performed new analyses of serologic and vaccine efficacy (VE) data from two randomized vaccine-controlled trials of the *Shigella sonnei*–*Pseudomonas aeruginosa* recombinant exoprotein A (rEPA) conjugate conducted in young adults and children aged 1–4 years in Israel. Adults received either *S. sonnei–r*EPA (*n* = 183) or control vaccines (*n* = 277). Children received the *S. sonnei–r*EPA conjugate (*n* = 1384) or *S. flexneri* 2a–rEPA conjugate (*n* = 1315). VE against culture-proven shigellosis was determined. Sera were tested for IgG anti–*S. sonnei* LPS antibodies. We assessed the association of various levels of IgG anti–*S. sonnei* LPS antibodies with *S. sonnei* shigellosis risk using logistic regression models and the reverse cumulative distribution of IgG levels.

**Results:**

Among adults, four vaccinees and 23 controls developed S. *sonnei* shigellosis; the VE was 74% (95% CI, 28–100%). A threshold of ≥1:1600 IgG anti–*S. sonnei* LPS titre was associated with a reduced risk of *S. sonnei* shigellosis and a predicted VE of 73.6% (95% CI, 65–80%). The IgG anti–*S. sonnei* LPS correlated with serum bactericidal titres. In children, a population-based level of 4.5 ELISA Units (EU) corresponding to 1:1072 titre, predicted VE of 63%, versus 71% observed VE in children aged 3–4 years. The predicted VE in children aged 2–4 years was 49%, consistent with the 52% observed VE.

**Conclusion:**

Serum IgG anti–*S. sonnei* LPS threshold levels can predict the degree of VE and can be used for the evaluation of new vaccine candidates.

## Introduction

*Shigella* is a leading cause of diarrhoea and is associated with a substantial burden globally. In the low- and middle-income countries, >250 million cases of shigellosis and >212 000 deaths are estimated to occur annually, mostly in children [1]. In these settings, *Shigella flexneri* accounts for most cases [2], whereas in the high-income countries, *Shigella sonnei* is the most common [1,3].

*Shigella* infection confers, although of limited duration, serotype-specific immunity, pointing to the O-specific polysaccharide (O-SP) as the protective antigen [[3], [4], [5]].

No licensed vaccine against *Shigella* is currently available, but several vaccine candidates have been tested in clinical trials in the last decades, including O-SP–based conjugate vaccines [6]. The first generation of *Shigella* conjugate vaccines was developed at the U.S. National Institutes of Health and evaluated in double-blind randomized vaccine-controlled trials in Israel [7,8]. A randomized clinical trial (RCT) of the *S. sonnei–*rEPA conjugate among military recruits [7] showed significant protection against *S. sonnei* shigellosis; the vaccine efficacy (VE) was 74% (p 0.007). A subsequent RCT of *S. sonnei* and *S. flexneri* 2a O-SP–rEPA conjugates among children aged 1–4 years showed 71% VE (p 0.043) of the *S. sonnei*–rEPA conjugate in the children aged 3–4 years but not in younger ones. Immunogenicity and efficacy were age-related and correlated [8].

The second generation of *Shigella* conjugates and other subunit vaccines are presently in clinical development [[9], [10], [11], [12]].

The identification of an immunological correlate of protection for shigellosis is essential for the development and licensure of *Shigella* vaccines and can inform Go/No-Go decisions in the evaluation of these vaccines.

Accumulated evidence indicates that serum IgG antibodies against *Shigella* lipopolysaccharide (LPS) can be a mechanistic immunological correlate of protection [13,14].

Here, leveraging serology and efficacy data from the mentioned above RCTs, we evaluated whether defined serum IgG anti–*S. sonnei* LPS levels induced by the *S. sonnei–*rEPA vaccine could predict the degree of protection against homologous *Shigella* infection.

## Methods

### Study design and population

#### New analyses of data from the RCT in young adults

For a detailed description of the trial, please see supplementary material. We reanalysed the serological results of IgG anti–*S. sonnei* LPS levels in vaccinees and controls of units A–C, in which *S. sonnei* shigellosis cases occurred 70–155 days post-vaccination [15].

Pre-vaccination (baseline, day 0) and post-vaccination (day 17 [range, 14–23] and day 137 [range, 97–170]) sera were obtained and tested using ELISA as described [7,15].

The results were expressed as endpoint titres; geometric mean titres (GMTs) and ≥4-fold increases in titre post-vaccination over baseline were calculated [7]. We assessed the associations of various titres of IgG anti–*S. sonnei* LPS as potential thresholds, with the incidence of culture-proven *S. sonnei* shigellosis and whether they could predict the VE of the *S. sonnei–*rEPA conjugate.

Serum bactericidal activity (SBA) was examined in 20 remaining archived sera of vaccinees on day 17 post-vaccination as described [9].

We estimated the potential threshold of serum IgG anti–*S. sonnei* LPS that might be associated with *S. sonnei* shigellosis incidence using multiple approaches to verify the robustness of our findings, especially given the small number of shigellosis incident cases during the trial.

The primary analysis included bivariate and multivariable logistic regression models to assess the Prentice criteria for a valid immunological substitute endpoint [16] (please see supplementary material). The incidence of culture-proven *S. sonnei* shigellosis was the dependent variable. The independent variables comprised vaccination status (vaccine vs. control) and serum IgG levels expressed in titres (1:800, 1:1200, 1:1600, 1:1800, 1:2000, and 1:3200), or ≥4-fold rise in titre on day 17. We hypothesized that the serum IgG levels will blunt the association between vaccination and shigellosis, demonstrating that just antibodies were those that protect against the disease. The multivariable model also included an interaction term between vaccination and antibody levels and adjusted for baseline antibody titres as a putative confounder [17,18]. Firth's correction was used in the regression model [19] given the small number of shigellosis cases.

Sera on day 17 post-vaccination were obtained from 371 of 450 (82%) participants. Therefore, we employed the predictive mean matching multiple imputation method using the observed information on vaccination status and baseline serum IgG anti–*S. sonnei* LPS levels to replace the missing values of day 17 antibody level. Multiple imputations were deemed accurate because the sample in this RCT was homogenous regarding age, sex, and risk of exposure.

Mantel-Haenszel analysis was employed to examine the effect of vaccination on disease in stratification by levels of antibodies ≥1:1600 or <1:1600, 17 days post-vaccination.

We also used the method developed by Siber et al. [20] to estimate the level of IgG titre that defines a threshold correlate of the protection model assuming perfect/complete versus zero protection against the disease [20] (*S. sonnei* shigellosis). We compared the VE estimated by the calculated immunological threshold (VE_IM_) with the observed VE calculated using vaccination status only, to evaluate the accuracy of the threshold correlate of the protection model.

The VE_IM_ formula is as follows:

$$VE_{\mathrm{IM}}=1-(\frac{Proportion\;of\;vaccinated\;individuals\;having\;a\;level\;of\;IgG\;anti-S.sonnei\;LPS\;below\;the\;threshold}{Proportion\;of\;unvaccinated\;individuals\;\left(controls\right)\;with\;a\;level\;of\;IgG\;anti-S.sonnei\;LPS\;below\;the\;threshold})\times 100$$

Consistency between VE_IM_ and the observed VE supports the utility of using a simple threshold correlate of the protection model [20,21]. The Taylor series [21] was used to calculate the 95% CIs for the predicted VEs.

We also used a nonparametric method [22] to estimate IgG thresholds corresponding to any specified risk, including zero risk, to provide a 95% CI for the estimated threshold levels. This method accounts for missing day 17 serology data by inverse probability of observation weights and accounts for loss to follow-up of some participants.

Data were analysed using the SPSS Statistics, version 28 (IBM Corp., Armonk, NY, USA), SAS Software, version 9.4 (SAS Institute Inc., Cary, NC, USA), and R version 4.1.0 (The R Foundation for Statistical Computing, Vienna, Austria).

#### New analyses of data from the RCT in children

For a detailed description of the trial, please see supplementary material. We applied the approach reported by Siber et al. [20] as described above in data analysis of the paediatric RCT [8], in which serological results were available for 10% of randomly selected participants. The paucity of sera did not allow individual-level assessment of the association between IgG anti–*S. sonnei* LPS and *S. sonnei* shigellosis. The VE_IM_ of the *S. sonnei*–rEPA conjugate was compared with the observed VE by age group.

#### Commutability of IgG anti–*S. sonnei* LPS measurement units in the two trials

In the RCT of young adults, IgG anti–*S. sonnei* LPS levels were expressed as endpoint titres, whereas in the paediatric RCT, they were expressed in EU related to a standard prepared at the U.S. National Institutes of Health from convalescent sera of *S. sonnei* shigellosis patients assigned 100 EU. To attain commutability between the two units, the conversion of the endpoint titres to EU (standing for a percentage of standard) was performed (Figs. S1 and S2).

## Results

**Fig. 1:**
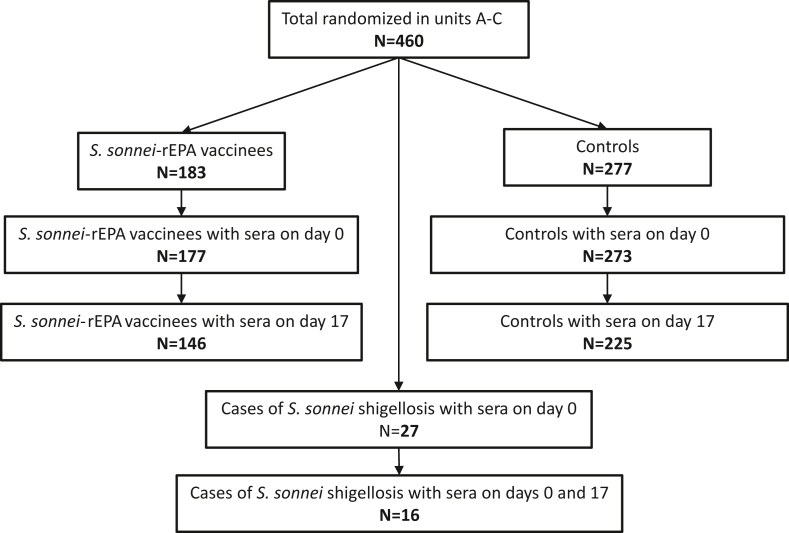
Flowchart of study participants.

Overall, 460 participants were randomized in units A–C in the young adults' trial, 183 and 277 in the *S. sonnei*–rEPA vaccine and control groups, respectively. Sera were obtained from 177 vaccinees and 273 controls on day 0 and from 146 vaccinees and 225 controls on day 17 (Fig. 1).

Overall, 27 participants developed *S. sonnei* shigellosis during the follow-up, 4 (2.2%) among recipients of S sonnei–rEPA vs. 23 (8.3%) among the control group, yielding a VE of 74% (95% CI, 28–100%; p 0.007) (Table S1 from [7]).

**Fig. 2:**
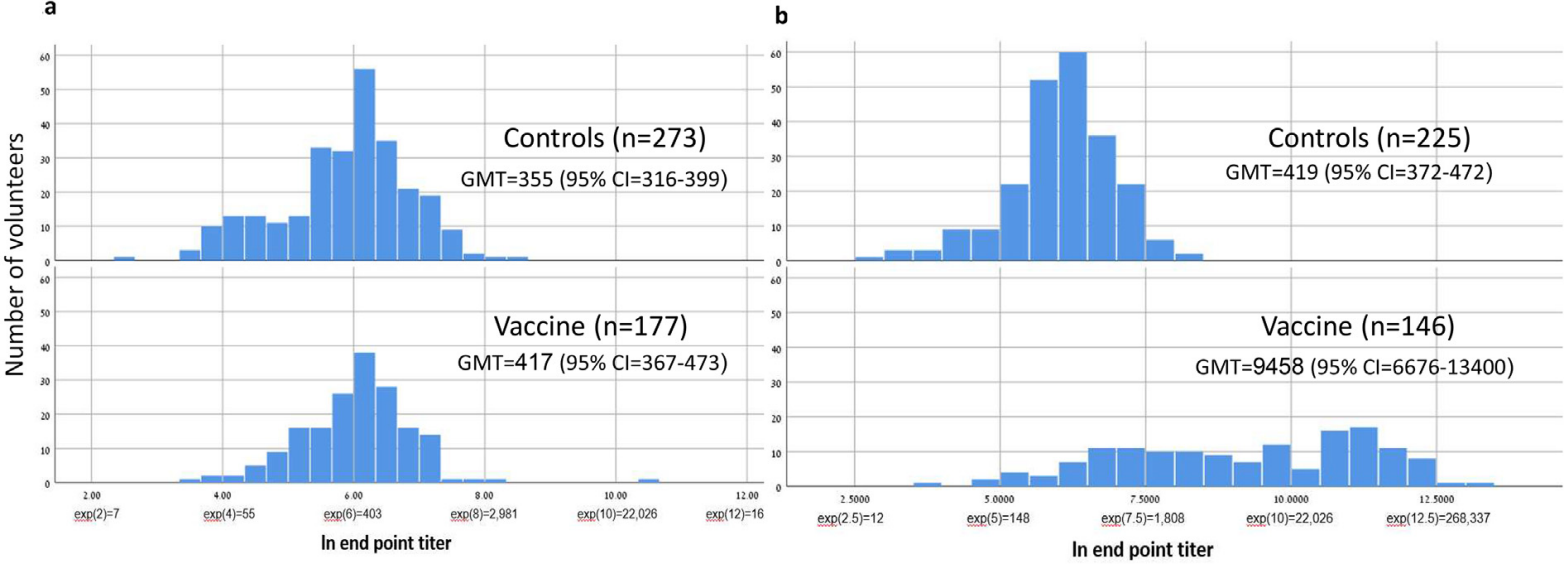
(a) Distribution of IgG anti–*S. sonnei* lipopolysaccharide among vaccinees and controls on day 0 (before vaccination). Number of volunteers (y axis) and ln endpoint titres (x axis) of controls and vaccines on day 0 (before vaccination); geometric mean titres (GMTs) (95% CI). (b) Distribution of IgG anti–*S. sonnei* lipopolysaccharide among vaccinees and controls on day 17 after vaccination (with serum samples available for 82% of volunteers). Number of volunteers (y axis) and ln endpoint titres (x axis) of controls and vaccinees on day 17 after vaccination; GMTs (95% CI).

Before vaccination, there was no significant difference in GMTs of IgG anti–*S. sonnei* LPS between the vaccinees and controls. On day 17, the GMT was approximately 20 times higher in vaccinees (GMT = 9458; 95% CI, 6676–13 400) than in controls (GMT = 419; 95% CI, 372–472) (p < 0.001) (Fig. 2).

Overall, 107 of 146 (73%) vaccinees showed a ≥4-fold rise in titre between days 0 and 17.

On day 17, the GMT of IgG anti–*S. sonnei* LPS was significantly lower among participants who subsequently developed *S. sonnei* shigellosis than in those without shigellosis (Table 1).

**Table 1:** IgG anti–*S. sonnei* lipopolysaccharide level 17 days after the administration of *S. sonnei*–rEPA or control vaccines in volunteers who subsequently developed or did not develop *S. sonnei* shigellosis: complete-cases analysis

Volunteers	N	GMT (95% CI)
Without shigellosis	355	1480 (1172–1849)
Subsequently developed *S*. *sonnei* shigellosis	16	735 (520–1035)
P		0.001

GMT, geometric mean titre.

Using the antibody distribution (Fig. 2), we selected several IgG anti–*S. sonnei* LPS cut-off levels as independent variables. The cutoffs 1:1600, 1:1800, and 1:2000 had the strongest association with *S. sonnei* shigellosis incidence. Having a titre of ≥1:1600 (7.4, the ln of 1600) was inversely related to *S. sonnei* shigellosis (OR = 0.13; 95% CI, 0.01–0.77; p 0.026). The results were similar when using imputed data for day 17 missing IgG titre results (Table 2, and Tables S2, S3) and when using ≥4-fold rise IgG anti–*S. sonnei* LPS titres (OR = 0.16; 95% CI, 0.01–0.89; p 0.047).

**Table 2:** Serum IgG anti–*S. sonnei* lipopolysaccharide at the cut-off 7.4 (ln 1600) on day 17 after vaccination and cases of *S. sonnei* shigellosis

IgG anti–*S.sonnei* LPS	No. without *S. sonnei* shigellosis	%	Cases of *S. sonnei* shigellosis	%	OR (95% CI)	p
Complete-cases analysis: individuals with available sera on day 17 post-vaccination						
IgG < ln 1600	237	67	15	94	Reference	0.026
IgG ≥ ln 1600	118	33	1	6	0.13 (0.01–0.77)	
Total	355	100	16	100		
Imputed dataset: multiple imputation for missing values of sera on day 17 postvaccination^a^						
IgG < ln 1600	288	66.5	25	92.6	Reference	0.039
IgG ≥ ln 1600	145	33.5	2	7.4	0.15 (0.025–0.910)	
Total	433	100	27	100		

a After multiple imputation (predictive mean matching method).

A multivariable logistic regression model (complete-case analysis and using an imputed data set) that included vaccination status and IgG anti–*S. sonnei* LPS levels as predictors and *S. sonnei* shigellosis as the dependent variable confirmed our hypothesis that anti–*S. sonnei* LPS levels blunt associations between vaccination and shigellosis (Table S4), thus supporting the inference that the efficacy of *S. sonnei* conjugate is mediated via the serum IgG anti–*S. sonnei* LPS. The results were similar when using a ≥4-fold increase in IgG anti–*S. sonnei* LPS on day 17 as the independent variable (Table S5). The results of Mantel-Haenszel stratified analysis by day 17 IgG anti–*S. sonnei* LPS levels were similar (Table S6).

The estimated probability of disease as a function of IgG anti–*S. sonnei* LPS titres from logistic regression models showed good separation between participants with and without shigellosis at a titre of 1:1600 (Fig. S3).

Using the nonparametric method reported by Donovan et al. [22] to estimate IgG threshold levels corresponding to any specified risk, including zero risk, while accounting for missing data, showed that overall there was a decreasing risk of disease for all participants with IgG anti–*S. sonnei* LPS increasing threshold levels (Fig. S4).

### Reverse cumulative distribution of antibody levels approach

The reverse cumulative distribution of ln antibody levels on day 17 post-vaccination showed that the threshold of 7.36 (ln of endpoint titre 1:1600) was consistent with the 74% (95% CI, 28–100%) observed VE in this trial (Table S7 and Fig. S5).

### SBA

IgG anti–*S. sonnei* LPS and SBA titres in day 17 post-vaccination sera of 20 *S. sonnei*–rEPA vaccinees were highly correlated (*r* = 0.9; p < 0.001) (Fig. S6).

### Putative antibody threshold of protection in children

**Fig. 3:**
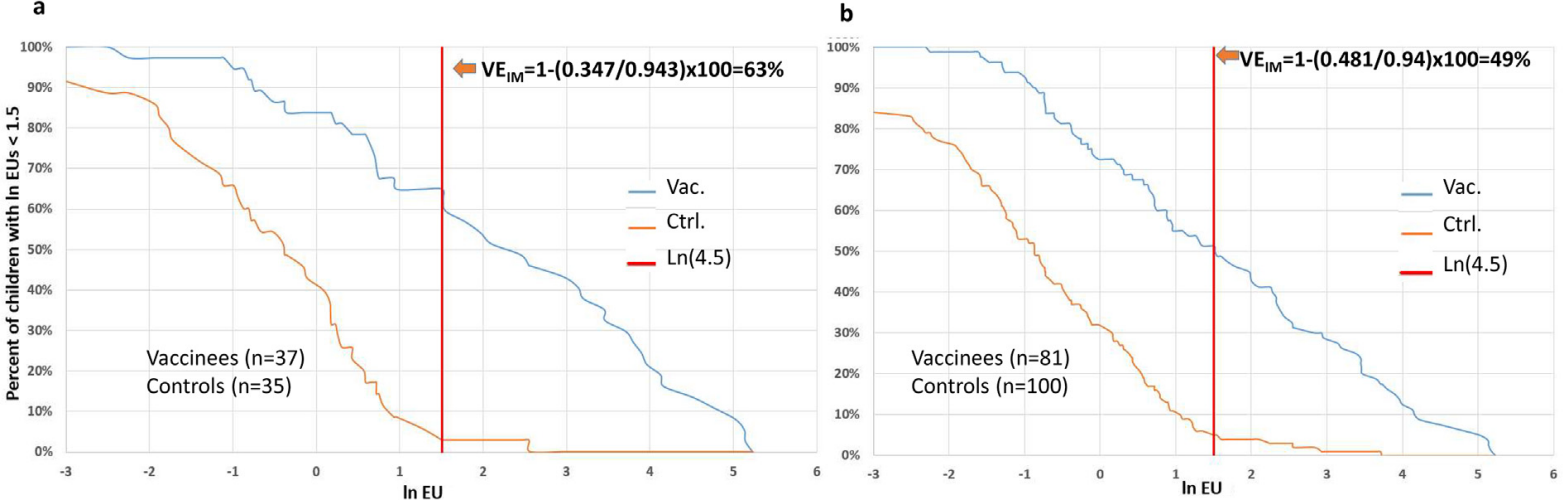
(a) Reverse cumulative distribution of ln IgG EU levels in children aged 3–4 years, vaccinees (blue curve) and controls (brown curve), and vertical line (red) illustrating the 1.5 (ln 4.5 EU) threshold location. (b) Reverse cumulative distribution of ln IgG EU levels in children aged 2–4 years, vaccinees (blue curve) and controls (brown curve), and vertical line (red) illustrating the 1.5 (ln 4.5 EU) threshold location. VE_IM_, immunological/predicted vaccine efficacy.

The VE of the *S. sonnei*–rEPA conjugate at 2 years post-vaccination was 71.1% (95% CI, 4.43–92.0%) in children aged 3–4 years, but it was lower and not statistically significant in the age groups 2–3 years and 1–2 years. A pooled analysis of children aged 2–4 years yielded a VE of 51.6% (95% CI, 1.0–76.0%) (Table S8). The VE_IM_ could be assessed on the basis of the proportion of children vaccinated with the *S. sonnei–*rEPA conjugate and control vaccine who had IgG anti–*S. sonnei* LPS levels below the different candidate thresholds. These results are shown for two age groups: 3–4 and 2–4 years (Table S9 and Fig. 3). The cut-off of 1.5 (ln of 4.5 EU) yielded a VE_IM_ of 63% (95% CI, 42–76%), slightly lower than the 71% observed VE for children aged 3–4 years. The same cut-off predicted a VE_IM_ of 49% (95% CI, 35–59%), consistent with the 52% observed VE for the children aged 2–4 years (Tables S8 and S9).

### Commutability between ELISA IgG endpoint titres and EU

The orthogonal linear regression analysis and fitted equation used for conversion indicated that the value of 1:1600 endpoint titre measured 17 days post-vaccination (exp of ln 7.38), the threshold associated with 74% protection in young adults, corresponded to the value of 6.6 EU (95% CI, 5.7–7.4) [exponent (exp) of ln 1.89 (95% CI, 1.74–2.01)] (Fig. S2 and Tables S10 and S11).

The 4.5 EU (exp of ln 1.5) measured ≥2 weeks post-vaccination, at a median time of 18.5 weeks after vaccination, and associated with 52% protection in children aged 2–4 years corresponded to 1:1072 titre (95% CI, 1:937 to 1:1226).

## Discussion

The main findings of our study are the identified protective threshold levels of IgG anti–*S. sonnei* LPS against *S. sonnei* shigellosis in young adults and children. In the RCT comprising adults, a threshold of 1:1600 (∼6.6 EU) IgG anti–*S. sonnei* LPS titre at day 17 post-vaccination reduced the risk of shigellosis and predicted an *S. sonnei–r*EPA VE_IM_ of 73.6%, consistent with the observed 74% VE. A post-vaccination level of 4.5 EU of IgG anti–*S. sonnei* showed 63% VE_IM_ in children aged 3–4 years, slightly lower than the observed 71% *S. sonnei*–rEPA conjugate VE and VE_IM_ of 49% similar to the observed 52% VE in children aged 2–4 years.

The findings from this re-analysis of data from *S. sonnei*–rEPA conjugate RCTs corroborate with results from our observational studies regarding the pivotal role of pre-existing serum IgG anti–*S. sonnei* LPS in the protection against the homologous disease [23,24] and collectively highlight the usefulness of IgG anti-LPS as a correlate of protection [13] that can foster the development and licensure of *Shigella* vaccines.

In the RCT comprising young adults, where we used individual-level data, confirming the Prentice criteria [16], we found that the full effect of the vaccine is mediated via IgG anti–*S. sonnei* LPS by documenting that the protection by the *S. sonnei*–rEPA vaccine is annulled after adjusting for the day 17 post-vaccination IgG anti–*S. sonnei* LPS in the logistic regression model. This was confirmed by the stratified Mantel-Haenszel analysis.

In children, we used the population-based predicted VE_IM_ following the methodology employed by Siber et al. [20] to identify the minimal protective antibody levels that supported the licensure of new pneumococcal and meningococcal vaccines without conducting large efficacy trials [20,21].

The conversion of endpoint titres to EU enabled the identification of a ‘dose-response’ relationship between the antibody levels and protection levels across the two trials. The 6.6 EU threshold level (corresponding to 1600 endpoint titre) was measured 17 days post-vaccination in young adults and predicted 73.6% VE against culture-proven *S. sonnei* shigellosis occurring 71–155 days post-vaccination. The 4.5 EU threshold level was identified at the population level in children aged 2–4 years at a median time of 18.5 weeks after vaccination, whereas more than half of the cases of *S. sonnei* shigellosis occurred in the second year of follow-up. At this time after vaccination, the 4.5 EU threshold yielded a VE_IM_ of 49%, consistent with the observed VE in this merged age group (52%).

We found a strong significant correlation between IgG anti–*S. sonnei* LPS of vaccinees and SBA. The antibody-mediated complement-dependent bacteriolysis was proposed as the most plausible mechanism by which IgG anti–*S. sonnei* LPS can protect against *Shigella* infection [8,25,26]. ELISA for serum IgG antibodies against *Shigella* LPS is an easy and inexpensive assay which can be practically applied to large RCTs of candidate vaccines. The strong correlation between vaccine-induced IgG anti-LPS levels and SBA titres indicates that the functional activity of ELISA-measured binding antibodies can be inferred.

The main strength of our study is the utilization of data from the only two available *S. sonnei*–rEPA conjugate field efficacy RCTs to link between vaccination, vaccine-induced antibody level, and homologous shigellosis in the two studies and populations. The individual-level data in the RCT comprising adults enabled the assessment of a protective *Shigella* LPS antibody threshold using multiple analytical approaches that yielded consistent findings. Moreover, we were able to link between serum IgG anti-LPS and SBA, thus providing mechanistic insights for protection.

Our study has limitations, including the lack of day 17 post-vaccination sera in 18% of the adult RCT volunteers and the availability of sera only for 10% of the participants in the paediatric RCT, the latter allowed only for using the reverse cumulative distribution analytical approach in children. Lastly, the number of *S. sonnei* shigellosis cases was small. To address these limitations, we used several analytical approaches, including Firth's correction, in logistic regression models and multiple imputations for missing values on day 17 post-vaccination sera, which yielded consistent results.

There is a need to harmonize internationally between the ELISA assays employed and generate an International Standard Serum. This will enable assay results to reach a maximum external validity for the threshold levels of serum IgG antibodies to *Shigella* LPS associated with protection [27].

The findings of this study on serum IgG anti–*S. sonnei* LPS as a correlate of protection, including putative thresholds predicting field VE, are important in the regulatory process of licensure of the candidate *Shigella* tetravalent conjugate and other injectable subunit O-SP–based vaccine candidates [27].

## Author contributions

Conceptualization and design: D.C., C.A.M., R.S., K.M., S.A., P.B.G.; writing (review and editing): D.C., C.A.M., R.S., K.M., S.A., P.B.G.; critical review of statistical methodology and support: P.B.G.; laboratory methodology and support: R.S., A.B., S.M.-S., V.A., N.F.; analysis of data: S.G., T.Z.B. All authors reviewed and finalized the manuscript.

## Transparency declaration

The study was supported by Investment ID OPP1195433 from 10.13039/100000865Bill & Melinda Gates Foundation, USA. The authors declare that they have no conflicts of interest.
