# Supplementary material for: Threshold protective levels of serum IgG to Shigella lipopolysaccharide: re-analysis of Shigella vaccine trials data
Source: Clin Microbiol Infect. 2023 Mar;29(3):366–71. doi: 10.1016/j.cmi.2022.10.011 (PMC9993342; doi:10.1016/j.cmi.2022.10.011)
Supplement: Multimedia component 1 [file mmc1.docx]

Supplementary Material

Page Contents

1. Contents

3. Background information on the efficacy trial of the *S. sonnei*-rEPA conjugate in young adults

in Israel

4. Background information on the efficacy study of the *S. sonnei*-rEPA in children in Israel

5. Supplementary methods

6. Table S1: Cases of *S. sonnei* shigellosis in vaccinees and in controls and vaccine efficacy (VE) in young adults

7. Table S2: Associations of IgG anti-*S. sonnei* LPS on day 17 post-vaccination using

various threshold levels with the risk of *S. sonnei* shigellosis complete case-analysis

8. Table S3: Associations of IgG anti-*S. sonnei* LPS on day 17 post-vaccination using various threshold levels with the risk of *S. sonnei* shigellosis (after multiple imputation)

9. Table S4: Logistic regression analysis of the effect of vaccination and of IgG anti-*S.sonnei* LPS equal or higher than the threshold of 1:1600 titer on the risk of *S. sonnei* shigellosis

10. Table S5: Logistic regression analysis* on the effect of vaccination and IgG anti *S. sonnei* LPS rise

in titer on the risk of *S. sonnei* shigellosis

10. Table S6: The effect of vaccination on *S. sonnei* shigellosis – stratified analysis by serum IgG anti *S. sonnei* LPS levels

11. Table S7: Reverse cumulative distribution of ln IgG antibody levels to S. sonnei LPS in young adults vaccinated with the *S. sonnei*-rEPA conjugate and control vaccines/placebo

11. Table S8: Age-dependent *S. sonnei*-rEPA conjugate vaccine efficacy (VE_OBS_) in the Pediatric Clinical Trial in Israel at end of study (2 years after vaccination)

12. Table S9: Reverse cumulative distribution of ln IgG antibody levels to *S. sonnei* LPS in children vaccinated with the *S. sonnei*-rEPA conjugate and *S. flexneri* 2a-rEPA conjugate (control vaccine) 2 weeks or more after receiving the second dose of vaccine

13. Table S10: Orthogonal regression equation: y =-5.17+0.957*x

13. Table S11: Orthogonal regression equation: y =5.4+1.044*x

14. Figure S1: Orthogonal regression (Deming regression with variance ratio 1) of 6 control sera in 8 double dilutions and NIH standard to convert results from NIH protocol (in EU) to TAU protocol (in EU)

15. Figure S2: Orthogonal regression (Deming regression with variance ratio 1) to convert results

from endpoint titers to EUs

16. Figure S3a: IgG anti-*S. sonnei* LPS level (95% CI) 17 days after vaccination (independent variable)

and probability of disease in the study population with serum samples

16. Figure S3b: Fitted disease probabilities for vaccine and control groups after accounting for the

missing IgG data on day 17 by multiple imputation

17. Figure S4: Threshold of risk inference (weighted analysis)

18. Figure S5: Reverse cumulative distribution of ln IgG end point titers in vaccinees (blue curve)

and placebo recipients (brown curve) and vertical line (brown) illustrating the 7.4

(ln 1600 endpoint titer) threshold location

19. Figure S6: Correlation between IgG anti-*S.sonnei* LPS and SBA in vaccinees 17 days after

vaccination

20. Legend for figures S1-S6

Background information on the efficacy trial of the *S. sonnei*-rEPA conjugate in young adults in Israel (Cohen D. et al. Lancet 1997).

A double-blind randomized vaccine-controlled trial was conducted to assess the efficacy of the *S. sonnei-*rEPA conjugate vaccine and of an oral, live-attenuated *Escherichia coli*/*S flexneri* 2a (EcSf2a-2) hybrid vaccine among Israeli military recruits at high risk of exposure to *Shigella* during training under field conditions. The design of the efficacy study was described in details elsewhere (Cohen D et al. Lancet 1997). Briefly, volunteers were randomized to three vaccine groups to receive one injection of *S. sonnei*-rEPA and four doses of oral placebo; four oral doses of the EcSf2a-2 control vaccine and one injection of saline (placebo); or one injection of meningococcal tetravalent control vaccine and four doses of oral placebo.

During 2.5 to 7 months of surveillance overlapping the field training schedules, cases of culture-proven *S. sonnei* shigellosis occurred in 4 of the 7 participating military units (units A, B, C, and D). These four units included 787 volunteers: 312 received the *S sonnei*-rEPA conjugate, 316 received the EcSf2a-2 vaccine, and 159 received the meningococcal control vaccine.

The efficacy of the *S. sonnei*-rEPA was determined by comparing the incidence of culture-proven *S. sonnei* shigellosis in the *S. sonnei*-rEPA vaccine group with that of the recipients of the *EcSf*2a-2 and meningococcal vaccines which served as control group. This was done in units A-C in which cases of *S. sonnei* shigellosis occurred 70 to 155 days after vaccination yielding the vaccine efficacy of 74% (95% CI: 28-100). Units A-C were the target of the new analyses described in the present article linking between the serological and efficacy data to derive the putative threshold of IgG anti-*S. sonnei* LPS protective levels against *S. sonnei* shigellosis.

As also described in Cohen D. et al. Lancet 1997, in Unit D the cases of *S. sonnei* shigellosis occurred 1-17 days after vaccination, at a time earlier than needed for the vaccine to induce the optimal immune response. Nevertheless, serological data reported in the same article for unit D only showed that the IgG anti-*S. sonnei* LPS response among vaccinees correlated with a lower risk of *S. sonnei* shigellosis.

Background information on the efficacy study of the *S. sonnei*-rEPA in children in Israel (Passwell J. et al. Vaccine 2010)

A double-blinded, randomized and vaccine-controlled phase III evaluation of *S. sonnei* and *S. flexneri* 2a O-SP–rEPA conjugates delivered by intramuscular (IM) injections six weeks apart, into healthy one to four years old children, was conducted in Israel between May 1^st^ 2003 to January 31^st^, 2008.

Overall, 1433 children received *S. sonnei-*rEPA and 1366 received *S. flexneri* 2a-rEPA and 2699 (96.4%) completed the two-year follow-up after vaccination. Stool cultures were obtained for each episode of acute diarrhea (≥3 loose stools/day or a bloody/mucous stool). Sera were taken randomly from 10% of the participants for IgG anti-LPS.

The vaccine induced IgG anti-LPS was the highest between two to ten weeks after vaccination (IgG anti-*S. sonnei* LPS Geometric Mean (GM)=12.9 EU in 24 random samples of the one to four years old children) and declined thereafter, but the GM of vaccinees remained 3.9-fold higher than of the controls at least 30 weeks after the second vaccine dose (p<0.01).

The numbers for *S. flexneri* 2a were too small for meaningful analysis of *S. flexneri* 2a- rEPA VE. The overall efficacy of the *S. sonnei* conjugate was 27.5% (95% CI:-16.9-54.0), but it reached 71.1% (95% CI:-4.43-92.0) (p=0.043) in the three to four years old. Immunogenicity and efficacy were age-related and correlated; GM EU of 1.40, 3.71 and 6.38 and VE values of 3.8%, 35.5% and 71.1% in the age groups 1-2, >2-3 and >3-4, respectively ^18^.

Supplementary methods

Bivariate and multivariable logistic regression models were used to evaluate the Prentice criteria for a valid immunological substitute endpoint (Prentice RL Stat Med 1989; ) in the reanalysis of the young adults’ RCT, namely: a) Protection against the clinical endpoint (*S. sonnei* shigellosis) is significantly related to having received the vaccine (*S. sonnei*-rEPA conjugate); b) The substitute endpoint (IgG anti-*S. sonnei* LPS) is significantly associated with vaccination; c) The substitute endpoint (IgG anti-*S. sonnei* LPS) is significantly related to the occurrence of the clinical endpoint (*S. sonnei* shigellosis); d) The full effect of the vaccine on the frequency of the clinical endpoint is explained by the substitute endpoint, as it lies on the sole causal pathway.

Table S1: Cases of *S. sonnei* shigellosis^*^ in vaccinees and in controls and vaccine efficacy (VE) in young adults (Cohen D et al. Lancet 1997).

| P value | Vaccine efficacy (95% CI) | Incidence rate | Cases of S. sonnei shigellosis* | Total randomized |  |
| --- | --- | --- | --- | --- | --- |
| 0.007 | 74% (28-100) | 8.3% | 23 | 277 | Controls |
|  |  | 2.2% | 4 | 183 | *S. sonnei*-rEPA conjugate vaccinees |

^*^Cases of disease occurred 71-155 days after immunization

Table S2: Associations of IgG anti-S. sonnei LPS on day 17 post-vaccination using various threshold levels with the risk of *S. sonnei* shigellosis (complete case-analysis):

| IgG anti-*S.sonnei* LPS | Without *S. sonnei* shigellosis | Percent | Cases of *S. sonnei* shigellosis | Percent | OR (95% CI) | P value |
| --- | --- | --- | --- | --- | --- | --- |
| IgG <ln 800 | 186 | 52% | 9 | 56% | Reference | 0.804 |
| IgG >ln 800 | 169 | 48% | 7 | 44% | 0.86 (0.30-2.40) |  |
| Total | 355 | 100% | 16 | 100% |  |  |
| IgG <ln 1200 | 219 | 62% | 13 | 81% | Reference | 0.185 |
| IgG >ln 1200 | 136 | 38% | 3 | 19% | 0.37 (0.08-1.24) |  |
| Total | 355 | 100% | 16 | 100% |  |  |
| IgG <ln 1600 | 237 | 67% | 15 | 94% | Reference | 0.026 |
| IgG >ln 1600 | 118 | 33% | 1 | 6% | 0.13 (0.01 to 0.77) |  |
| Total | 355 | 100% | 16 | 100% |  |  |
| IgG <ln 1800 | 240 | 68% | 15 | 94% | Reference | 0.027 |
| IgG >ln 1800 | 115 | 32% | 1 | 6% | 0.14 (0.01-0.80) |  |
| Total | 355 | 100% | 16 | 100% |  |  |
| IgG <ln 2000 | 246 | 69% | 15 | 94% | Reference | 0.047 |
| IgG >ln 2000 | 109 | 31% | 1 | 6% | 0.15 (0.01-0.86) |  |
| Total | 355 | 100% | 16 | 100% |  |  |
| IgG <ln 3200 | 258 | 73% | 15 | 94% | Reference | 0.080 |
| IgG >ln 3200 | 97 | 27% | 1 | 6% | 0.18 (0.01-1.02) |  |
| Total | 355 | 100% | 16 | 100% |  |  |
| IgG < 4-fold | 248 | 70% | 15 | 94% | Reference | 0.047 |
| IgG > 4-fold | 106 | 30% | 1 | 6% | 0.16 (0.01-0.89) |  |
| Total | 354 | 100% | 16 | 100% |  |  |
| IgG < 2-fold | 226 | 64% | 13 | 81% | Reference | 0.189 |
| IgG > 2-fold | 128 | 36% | 3 | 19% | 0.41 (0.09-1.36) |  |
| Total | 354 | 100% | 16 | 100% |  |  |

Table S3: Associations of IgG anti-*S. sonnei* LPS on day 17 post-vaccination using various threshold levels with the risk of *S. sonnei* shigellosis (after multiple imputation)

| IgG anti-S.s LPS | Without S. sonnei shigellosis | Percent | Cases of S. sonnei shigellosis | Percent | OR (95% CI) | P value |
| --- | --- | --- | --- | --- | --- | --- |
| IgG <ln 800 | 230 | 53% | 19 | 70% | Reference | 0.137 |
| IgG >ln 800 | 203 | 47% | 8 | 30% | 0.51 (0.21-1.24) |  |
| Total | 433 | 100% | 27 | 100% |  |  |
| IgG <ln 1200 | 267 | 62% | 23 | 85% | Reference | 0.054 |
| IgG >ln 1200 | 166 | 38% | 4 | 15% | 0.31 (0.09-1.02) |  |
| Total | 433 | 100% | 27 | 100% |  |  |
| IgG <ln 1600 | 288 | 67% | 25 | 93% | Reference | 0.039 |
| IgG >ln 1600 | 145 | 33% | 2 | 7% | 0.15 (0.03-0.91) |  |
| Total | 433 | 100% | 27 | 100% |  |  |
| IgG <ln 1800 | 291 | 67% | 25 | 93% | Reference | 0.043 |
| IgG >ln 1800 | 142 | 33% | 2 | 7% | 0.16 (0.03-0.94) |  |
| Total | 433 | 100% | 27 | 100% |  |  |
| IgG <ln 2000 | 297 | 69% | 25 | 93% | Reference | 0.05 |
| IgG >ln 2000 | 136 | 31% | 2 | 7% | 0.17 (0.03-1.00) |  |
| Total | 433 | 100% | 27 | 100% |  |  |
| IgG <ln 3200 | 313 | 72% | 26 | 96% | Reference | 0.053 |
| IgG >ln 3200 | 120 | 28% | 1 | 4% | 0.13 (0.02-1.03) |  |
| Total | 433 | 100% | 27 | 100% |  |  |
| IgG < 4-fold | 299 | 69% | 25 | 93% | Reference | 0.034 |
| IgG > 4-fold | 134 | 31% | 2 | 7% | 0.15 (0.03-0.86) |  |
| Total | 433 | 100% | 27 | 100% |  |  |
| IgG < 2-fold | 268 | 62% | 22 | 81% | Reference | 0.063 |
| IgG > 2-fold | 165 | 38% | 5 | 19% | 0.39 (0.14-1.06) |  |
| Total | 433 | 100% | 27 | 100% |  |  |

Table S4: Logistic regression analysis* of the effect of vaccination and of IgG anti-*S.sonnei* LPS equal or higher than the threshold of 1:1600 titer on the risk of *S. sonnei* shigellosis^$^

| Variable | Adjusted OR | 95% CI | P value |
| --- | --- | --- | --- |
| Model 1: Complete-cases analysis using dataset with available sera on day 17 post-vaccination ^$^ | | | |
| Vaccine vs. Placebo | 1.10 | 0.29 to 4.3 | 0.87 |
| Anti LPS IgG (day 17) > 1600 vs. lower level | 0.16 | 0.02 to 1.2 | 0.065 |
| Anti LPS IgG (day 0) | 1.38 | 0.82 to 2.36 | 0.223 |
| Model 2: Imputed dataset: multiple imputation for missing values of sera on day 17 post-vaccination^#^ | | | |
| Vaccine vs. Placebo | 0.531 | 0.12 to 2.3 | 0.397 |
| Anti LPS IgG (day 17) > 1600 vs. lower level | 0.262 | 0.02 to 2.8 | 0.262 |
| ln anti LPS IgG (day 0) | 0.895 | 0.60 to 1.3 | 0.590 |

*Firth’s corrections to adjust for the small number of outcome cases

^$^Adding an interaction term to the model did not change the results and the interaction was not significant p=0.9

^#^After multiple imputation (PMM method)

Model 1 (SAS-Firth’s correction):

Wald: Chi-Square=5.35, Pr > ChiSq=0.1479

Intercept: Estimate=-4.69, Wald Chi-Square =7.72, Pr > ChiSq=0.0055

95% CI: 95% Wald Confidence Limits

Model 2 (SPSS- multiple imputation):

Hosmer and Lemeshow Test: Chi-Square=2.0, Sig.=0.98

Intercept: B=-1.73, Sig.=0.150, OR=0.177 (0.02-1.88)

Method: Logistic Regression - Maximum Likelihood Estimation

Table S5: Logistic regression analysis^*^ on the effect of vaccination and IgG anti *S.*

| Variable | Adjusted OR | 95% CI | P value |
| --- | --- | --- | --- |
| Vaccine vs. Placebo | 0.883 | 0.138 to 3.358 | 0.872 |
| > 4-fold rise in IgG anti-*S. sonnei* LPS between days 0 and 17 | 0.173 | 0.008 to 1.776 | 0.155 |

*sonnei* LPS rise in titer on the risk of *S. sonnei* shigellosis

*Firth’s corrections to adjust for the small number of outcome cases

Wald: Chi-Square=3.22, Pr > ChiSq=0.1994

Intercept: Estimate=-2.79, Wald Chi-Square =95.18, Pr > ChiSq <0.0001

95% CI: Profile-Likelihood Confidence Intervals

| Unadjusted OR (95% CI)^$^ | P value* | *S. sonnei* shigellosis | Total | Vaccine status | IgG anti-*S. sonnei* LPS |
| --- | --- | --- | --- | --- | --- |
| Ref. | 1.0 | 13 (6.0%) | 216 | Placebo | IgG<ln 1600 |
| 1.02 (0.22-4.77) |  | 2 (5.6%) | 36 | Vaccine |  |
| Ref. | 1.0 | 0 (0%) | 9 | Placebo | IgG>ln 1600 |
| 0.09 (0.01-1.56) |  | 1 (0.9%) | 110 | Vaccine |  |
| Ref. | 0.11 | 13 (5.6%) | 225 | Placebo | Total |
| 0.34 (0.09-1.22) |  | 3 (2.6%) | 146 | Vaccine |  |

Table S6: The effect of vaccination on *S. sonnei* shigellosis – stratified analysis by serum IgG anti *S. sonnei* LPS levels

^*^ Fisher Exact test on 80% of the volunteers with samples on day 17; Breslow-Day test of homogeneity of the OR (p=0.09) (no interaction); ^$^Mantel-Haenszel adjusted OR vaccine vs. placebo 0.68 (95% CI 0.17-0.28) p=0.8

Table S7: Reverse cumulative distribution of ln IgG antibody levels to *S. sonnei* LPS in young adults vaccinated with the *S. sonnei*-rEPA conjugate and controls

| Group and cutoff | % Individuals with titers below threshold | | Predicted % vaccine  efficacy (95% CI)* |
| --- | --- | --- | --- |
|  | *S. sonnei*-rEPA | Controls |  |
| units A-C: |  |  |  |
| <6.7 (ln800) | 13.7% | 77.8% | 82.4 (73-88) |
| <7.1 (ln1200) | 20.5% | 90.7% | 77.3 (69-84) |
| <7.4 (ln1600) | 25.3% | 96.0% | 73.6 (65-80) |
| <7.5 (ln1800) | 26.7% | 96.4% | 72.3 (64-79) |
| <8.1 (ln3200) | 34.9% | 99.6% | 64.9 (56-72) |

*Predicted % vaccine efficacy (95% CI) = % Vaccinated with titers

below cutoff/% Controls (95% CI) with titers below threshold

Table S8: Age-dependent *S. sonnei*-rEpa conjugate vaccine efficacy (VE_OBS_) in the Pediatric Clinical Trial in Israel (Passwell et al. Vaccine 2010) at end of study (2 years after vaccination)

| Age group, years | Vaccine (cases/N) | Control (cases/N) | Vaccine efficacy | 95% CI | p |
| --- | --- | --- | --- | --- | --- |
| **>1-2** | 18/516 | 16/476 | 3.8% | 101.1-46.5 | 0.91 |
| **>2-3** | 8/497 | 12/481 | 35.5% | -56.4-73.4 | 0.33 |
| **>2-4** | 11/868 | 22/839 | 51.6% | 1.0-76.0 | 0.04 |
| **>3-4** | 3/371 | 10/358 | 71.1% | -4.43-92.0 | 0.04 |
| **All** | 29/1384 | 38/1315 | 27.5% | -16.9-54.0 | 0.18 |

Table S9: Reverse cumulative distribution of ln IgG antibody levels to *S. sonnei* LPS in children vaccinated with the *S. sonnei*-rEPA conjugate and *S. flexneri* 2a-rEPA conjugate (control vaccine) 2 weeks or more after receiving the second dose of vaccine

|  |  |  | | | | | | | | | |
| --- | --- | --- | --- | --- | --- | --- | --- | --- | --- | --- | --- |
|  |  | | | |  | | |  | |  |  |
| Age groups | Threshold | | | |  | | | % Individuals with IgG levels below threshold | | | Predicted % vaccine |
|  |  |  |  |  |  | | | *S. sonnei-*rEPA | | *S. flexneri* 2a-rEPA (control) | efficacy (95% CI) |
|  |  | | |  | | |  | | |  |  |
| 37-48 months: | <1.2 | | | ln3.3 | | | 35.1% | | 91.4% | | 61.6 (40-75) |
|  | <1.5 | | | ln4.5 | | | 35.1% | | 94.3% | | 62.7 (42-76) |
|  | <1.55 | | | ln4.7 | | | 37.8% | | 97.1% | | 61.0 (41-74) |
|  | <1.6 | | | ln5.0 | | | 40.5% | | 97.1% | | 58.3 (38-72) |
|  | <1.8 | | | ln6.0 | | | 43.2% | | 97.1% | | 55.5 (35-69) |
|  | <2 | | | ln7.4 | | | 45.9% | | 97.1% | | 52.7 (33-67) |
|  | <3 | | | ln20.1 | | | 56.8% | | 100.0% | | 43.2 (25-57) |
|  | <3.5 | | | ln33.1 | | | 67.6% | | 100.0% | | 32.4 (16-46) |
|  | <4 | | | ln54.6 | | | 78.4% | | 100.0% | | 21.6 (7-34) |
|  |  | |  | | | | |  | |  |  |
|  |  | | | |  |  | | | |  |  |
| 25-48 months: | <1.2 | | | | ln3.3 | 45.7% | | | | 91.0% | 49.8 (36-61) |
|  | <1.5 | | | | ln4.5 | 48.1% | | | | 94.0% | 48.8 (35-59) |
|  | <1.55 | | | | ln4.7 | 49.4% | | | | 95.0% | 48 (35-58) |
|  | <1.6 | | | | ln5.0 | 50.6% | | | | 95.0% | 46.7 (34-57) |
|  | <1.8 | | | | ln6.0 | 53.1% | | | | 96.0% | 44.7 (32-55) |
|  | <2 | | | | ln7.4 | 55.6% | | | | 96.0% | 42.1 (29-53) |
|  | <3 | | | | ln20.1 | 70.4% | | | | 99.0% | 28.9 (18-38) |
|  | <3.5 | | | | ln33.1 | 79.0% | | | | 99.0% | 20.2 (11-29) |
|  | <4 | | | | ln54.6 | 85.2% | | | | 100.0% | 14.8 (7-22) |

Table S10: Orthogonal regression equation: y =-5.17+0.957*x

| **Endpoint titer** | **ln end point titer (x)** | **Estimated ln EU (y)**  **95% CI** | **Estimated EU**  **95% CI** |
| --- | --- | --- | --- |
| 800 | 6.68 | 1.23 (1.1 - 1.36) | 3.4% (3% - 3.9%) |
| 1200 | 7.09 | 1.6 (1.5 - 1.76) | 5% (4.5% - 5.8%) |
| 1600 | 7.38 | 1.89 (1.74-2.01) | 6.6% (5.7%-7.4%) |
| 1800 | 7.50 | 2 (1.9 - 2.2) | 7.4% (6.7% - 9%) |
| 3200 | 8.07 | 2.6 (2.4 - 2.7) | 13.5% (11% - 14.9%) |

Table S11: Orthogonal regression equation: y =5.4+1.044*x

| **EU %** | **ln EU % (x)** | **Estimated ln end point titer (y)**  **95% CI** | **Estimated end point titer**  **95% CI** |
| --- | --- | --- | --- |
| 3.5 | 1.26 | 6.72 (6.58 - 6.85) | 825.7 (720.6 - 946) |
| 3.8 | 1.33 | 6.79 (6.65 - 6.92) | 888.3 (775.8 - 1017.1) |
| 4.1 | 1.41 | 6.87 (6.74 - 7.01) | 965.7 (843.8 - 1105.2) |
| 4.5 | 1.51 | 6.98 (6.84 - 7.11) | 1071.9 (936.9 - 1226.5) |
| 6 | 1.8 | 7.28 (7.14 - 7.42) | 1451.1 (1266.4 - 1662.7) |
| 7 | 1.94 | 7.43 (7.29 - 7.56) | 1679.5 (1463 - 1928) |

Figure S1: Orthogonal regression (Deming regression with variance ratio 1) of 6 control sera in 8 double dilutions and NIH standard to convert results from NIH protocol (in EU) to TAU protocol (in EU)


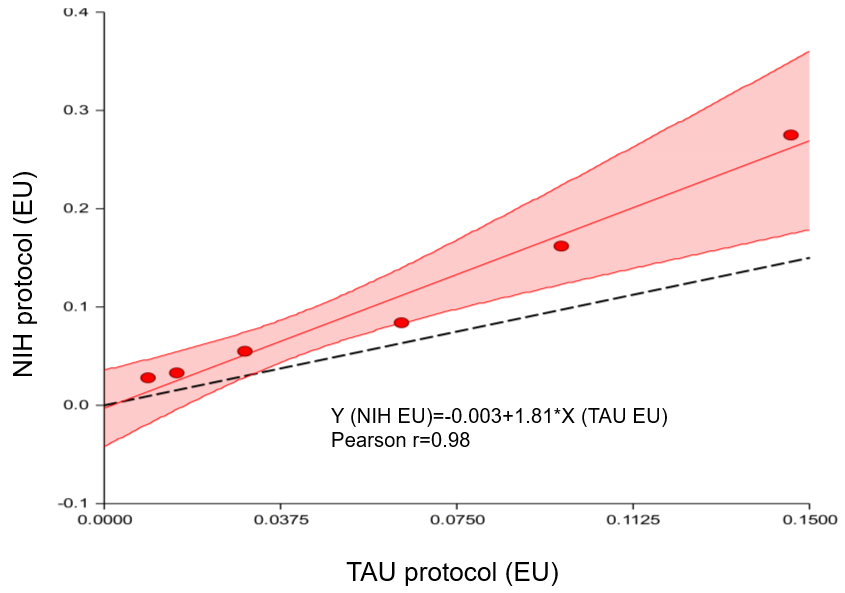


Figure S2: Orthogonal regression (Deming regression with variance ratio 1) to convert results from endpoint titers to EU


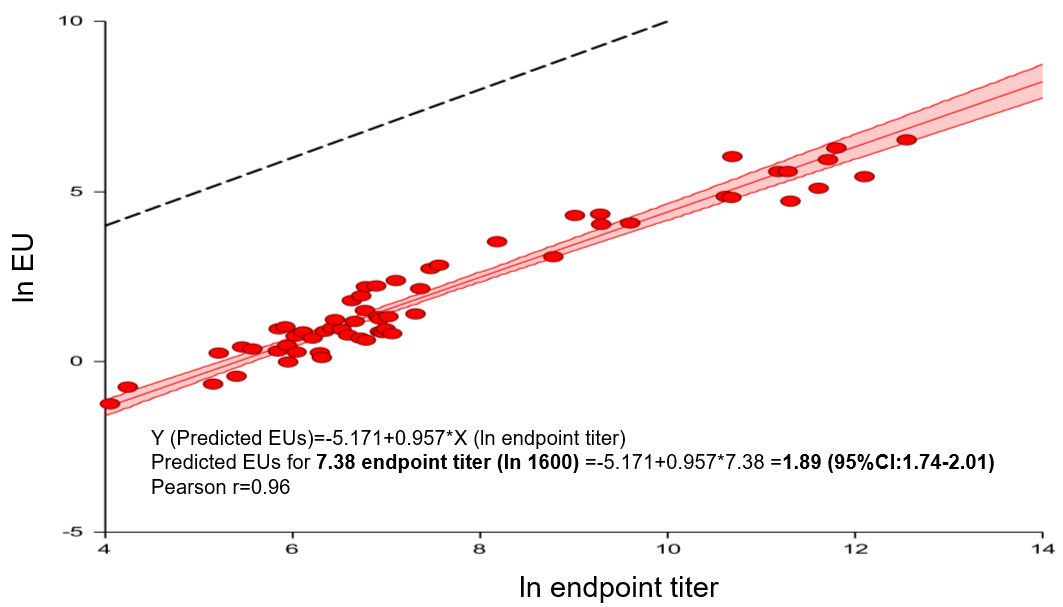


Figure S3a: IgG anti-*S. sonnei* LPS level (95% CI) 17 days after vaccination (independent variable) and probability of disease in the study population with serum samples (n=371)


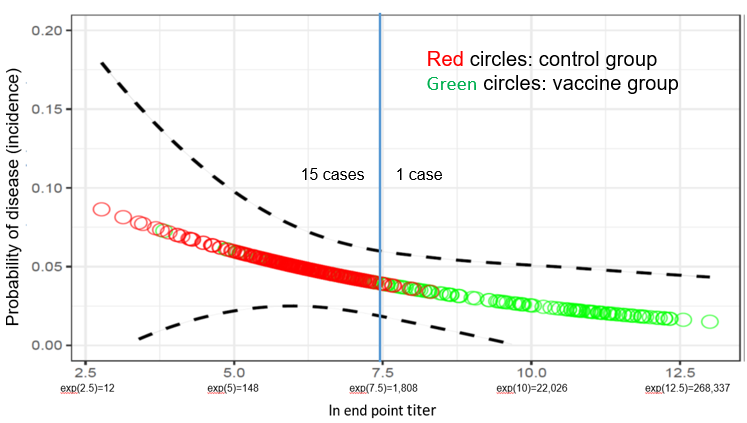


Figure S3b: Fitted disease probabilities for vaccine and control groups after accounting for the missing IgG data on day 17 by multiple imputation*


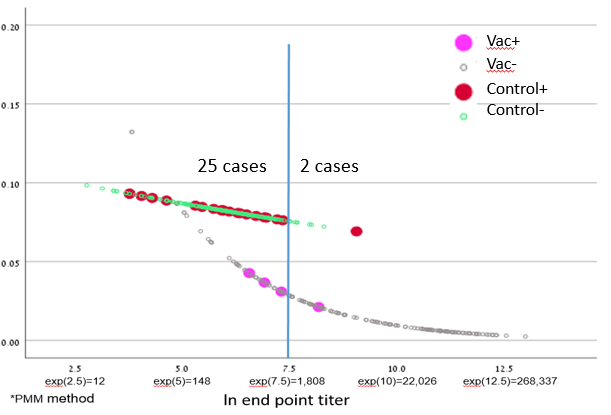


**Probability of disease (incidence)**

Figure S4: Threshold of risk inference (weighted analysis)


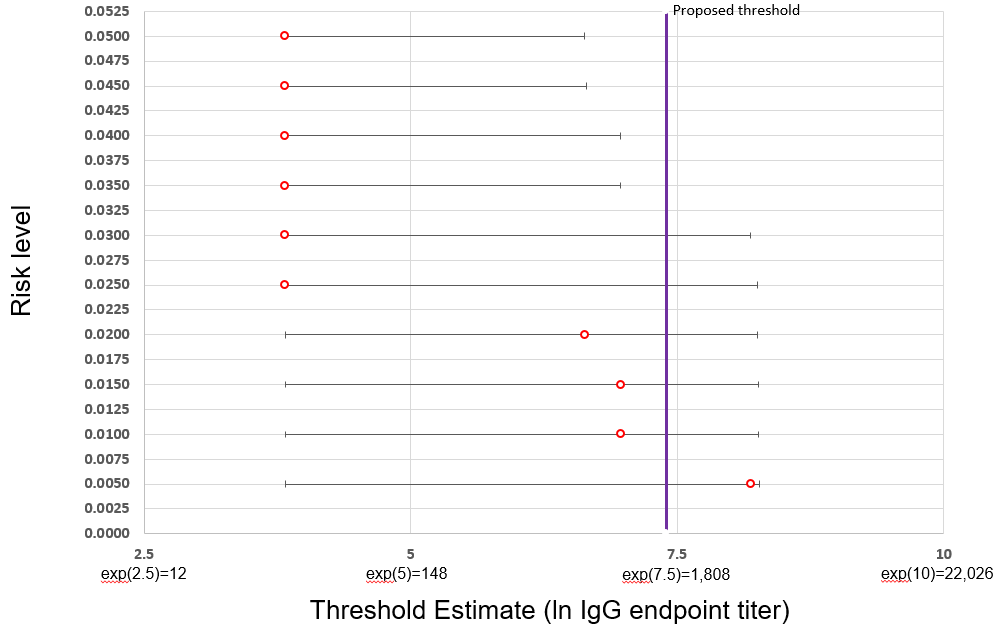


Figure S5: Reverse cumulative distribution of ln IgG endpoint titers in vaccinees (blue curve) and control vaccines recipients (brown curve) and vertical line (brown) illustrating the 7.4 (ln 1600 endpoint titer) threshold location


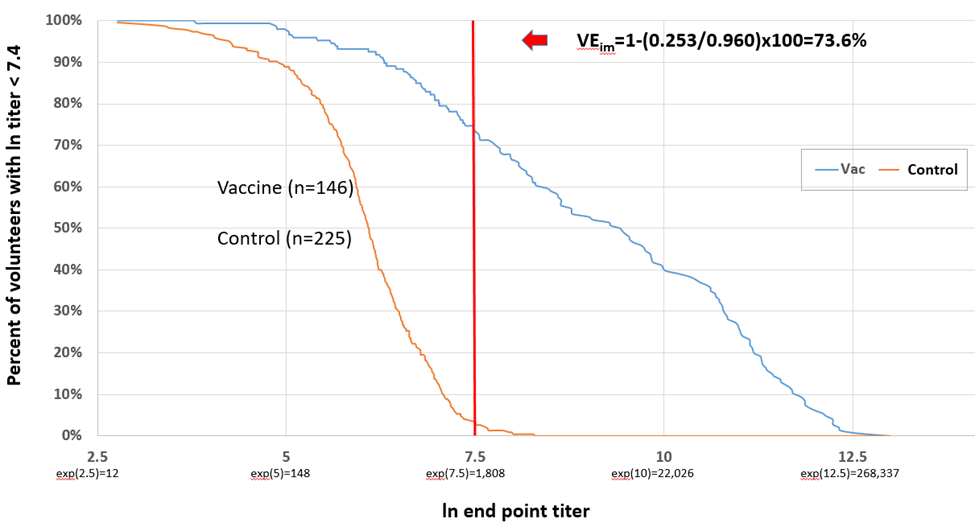


Figure S6: Correlation between IgG anti-S.sonnei LPS and SBA in vaccinees 17 days after vaccination


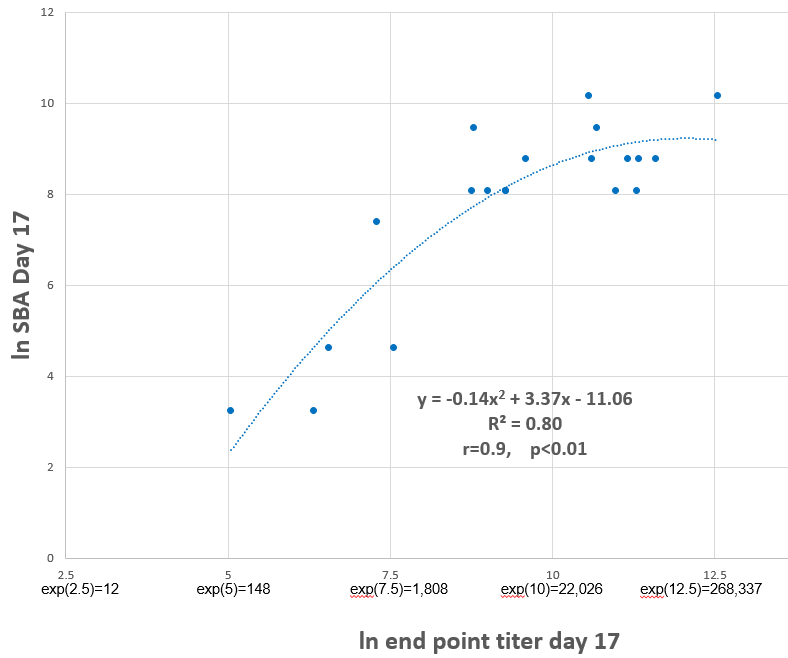


Legend for figures S1-S6

**Fig. S1**: Orthogonal regression plot (Deming regression with variance ratio 1) of 6 control sera in 8 double dilutions and NIH standard to convert results from NIH protocol (in EU) to TAU protocol (in EU)

**Fig. S2**: Orthogonal regression (Deming regression with variance ratio 1) to convert results from endpoint titers to EU; each red point represents the ln endpoint titer and the corresponding ln EU values; the equation Y (Predicted EU) =-5.171+0.957*X (ln endpoint titer) exemplifies the translation of endpoint titers to corresponding EU value.

**Fig. S3a**: IgG anti-*S. sonnei* LPS level 17 days after vaccination and probability of disease from logistic regression fit to all volunteers with serum samples (n=371); red circles represent controls; green circles represent vaccinees; interrupted lines indicate 95% CI

**Fig. S3b**: IgG anti-*S. sonnei* LPS level 17 days after vaccination and probability of disease from logistic regression fit to all vaccinees and controls separately after accounting for the missing IgG data on day 17 by multiple imputation (PMN method); green and red circles represent control arm recipients without or with *S. sonnei* shigellosis, respectively; grey and purple circles represent vaccinees without and with *S. sonnei* shigellosis, respectively; blue vertical line represents the proposed threshold (7.4 on the ln IgG end point titer on x axis)

**Fig. S4**: Ln IgG thresholds corresponding to specified risk levels (red circle) of all volunteers and 95% CI (black horizontal lines) for the estimated thresholds (Donovan et al. non-parametric method, weighted analysis)

**Fig. S5**: Reverse cumulative distribution of ln IgG end point titers in vaccinees (blue curve) and control arm recipients (brown curve) and vertical line (red illustrating the 7.4 (ln 1600 endpoint titer) threshold location corresponding to 74% observed VE

**Fig. S6**: Scatter plot illustrating the correlation between IgG anti-*S.sonnei* LPS and SBA in vaccinees 17 days after vaccination; each blue point represent the ln endpoint titers and the corresponding ln SBA titers
